# Essential Fatty Acid Deficiency in an Extremely Premature Infant With Intestinal Failure

**DOI:** 10.1097/PG9.0000000000000063

**Published:** 2021-04-12

**Authors:** Alvin P. Chan, Katie M. Strobel, Kara L. Calkins

**Affiliations:** From the * Division of Pediatric Gastroenterology, Hepatology and Nutrition, Department of Pediatrics, UCLA Mattel Children’s Hospital, Los Angeles, CA; † Division of Neonatology, Department of Pediatrics, UCLA Mattel Children’s Hospital, Los Angeles, CA.

## Abstract

Supplemental Digital Content is available in the text.

## INTRODUCTION

Essential fatty acid deficiency (EFAD) is a serious condition that can develop rapidly in premature infants who do not receive an adequate amount of lipid. The immediate consequences of EFAD include elevated transaminases, thrombocytopenia, poor wound healing, scaly dermatitis, hypertriglyceridemia (HTG), and growth failure. EFAD can also adversely affect brain development. In this case report, we discuss neonatal lipid management and its relationship with EFAD, HTG, and intestinal failure-associated liver disease (IFALD). Specifically, we present a premature infant with short bowel syndrome who developed a severe EFAD secondary to intravenous lipid emulsion (ILE) dose restriction. Parental consent was obtained to publish this case report.

## CASE

An infant born at 23 weeks gestational age was transferred at 2 weeks of age for renal failure, septic shock, and necrotizing enterocolitis. Prior to transfer, the infant was gavage fed and on noninvasive respiratory support. At 4 weeks of age, the infant underwent a laparotomy. The infant was found to have a bowel perforation with necrosis that was treated with peritoneal washouts, drains, antimicrobials, and bowel rest. The postoperative course was complicated by respiratory failure requiring ventilatory support for 3 weeks. The infant was also diagnosed with IFALD, postnatally acquired cytomegalovirus (CMV), chronic lung disease, bilateral intraventricular hemorrhages (grades 2 and 3), periventricular leukomalacia, and growth failure.

**FIGURE 1.**
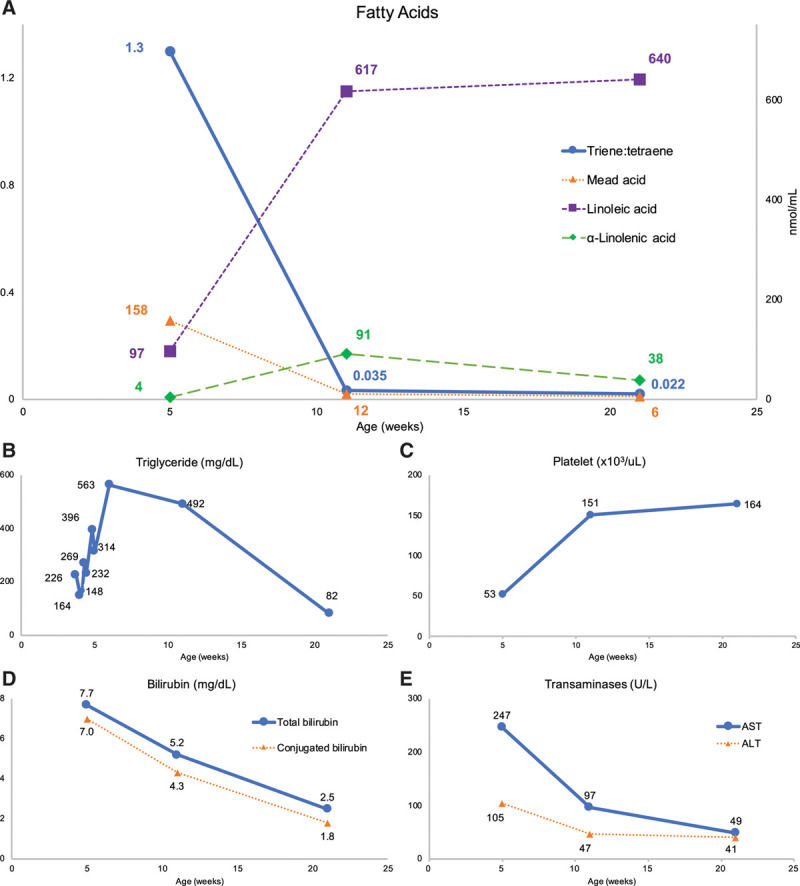
Graphical trends in nutritional and hepatobiliary markers. One hundred percent fish oil ILE was started at 5 wk of age. A) Fatty acids, (B) triglycerides (not all triglyceride concentrations are depicted), (C) platelet counts, (D) bilirubin levels, and (E) transaminases. ALT = alanine aminotransferase; AST = aspartate aminotransferase; ILE = intravenous lipid emulsion.

Parenteral nutrition was started on admission and advanced over the next several days (glucose infusion rate 13 mg/kg/min, amino acid 4 g/kg/d, and 84 kcal/kg/d). ILEs were held for 12 days after admission because of limited vascular access and fluid restriction due to renal failure. Thereafter, at 3.5 weeks of age, a mixed oil ILE with 15% fish oil (SMOFlipid; Fresenius Kabi, Bad Homburg, Germany) was prescribed at a dose of 0.5 g/kg/d for the next 4 days. At 5 weeks of age, the infant was given a single dose of 3.5 g/kg of this mixed oil ILE (equivalent to 0.5 g/kg/d) due to concern for HTG (range triglyceride, 148–563 mg/dL, Fig. 1). The infant remained nil per os.

At 5 weeks of age, the infant was diagnosed with a severe EFAD, hallmarked by a high triene:tetraene ratio (T:T) of 1.3, elevated transaminases, thrombocytopenia, poor healing, dry skin, and growth failure (Table 1). One hundred percent fish oil ILE (Omegaven; Fresenius Kabi, Bad Homburg, Germany) dosed at 1.5 g/kg/d was prescribed to treat the infant’s EFAD, HTG, and IFALD (Table 1). By 21 weeks of age, after 16 weeks of 100% fish oil ILE, the T:T and transaminases normalized and the conjugated bilirubin was <2 mg/dL (Table 1 and Fig. 1).

**TABLE 1.** Growth, nutrition, and laboratory parameters

Parameters	5 wk	11 wk	21 wk
Growth			
** **Weight, g (*z* score)	811 (–1.2)	1320 (–2.6)	3.025 (–2.4)
** **Length, cm (*z* score)	32 (–1.8)	34 (–4.0)	46 (–3.9)
** **Head circumference, cm (*z* score)	20.6 (–1.8)	24.5 (–4.5)	31 (–4.5)
Nutrition			
** **Parenteral nutrition, kcal/kg/d	88	109	99
** **Glucose infusion rate, mg/kg/min	13.6	16	13.6
** **Amino acid, g/kg/d	4	4	3.9
** **Lipid, g/kg/d	0.5*	1.5†	1.5†
** **Enteral nutrition, kcal/kg/d	0	0	0
Laboratory markers			
** **Aspartate aminotransferase, U/L	247	97	49
** **Alanine aminotransferase, U/L	105	47	41
** **Total bilirubin, mg/dL	7.7	5.2	2.5
** **Conjugated bilirubin, mg/dL	7.0	4.3	1.8
** **Platelet, ×10^3^/uL	53	151	164
** **Triglyceride, mg/dL	314	82	97
** **Triene:tetraene (reference 0.013–0.050)‡	1.3	0.035	0.022
** **Linoleic acid, nmol/mL (reference 1000–3300)‡	97	617	640
α-Linolenic acid, nmol/mL (reference 10–190)‡	4	91	38
** **Mead acid, nmol/mL (reference 3–24)‡	158	12	6
** **Arachidonic acid, nmol/mL (reference 110–1110)‡	22	27	24
** **Docosahexaenoic acid, nmol/mL (reference 10–220)‡	84	1000	739
** **Eicosapentaenoic acid, nmol/mL (reference 2–60)‡	6	1002	820

ILE = intravenous lipid emulsion.

*Mixed oil ILE with 15% fish oil.

†100% fish oil ILE.

‡Plasma fatty acid profiles performed at ARUP Laboratories (Salt Lake City, UT). Reference values based on published plasma fatty acid ranges (1).

At 20 weeks of age, the infant returned to the operating room for a laparotomy for lysis of adhesions, jejunal stricture resection, and jejunoileostomy. Parenteral nutrition was discontinued 4 weeks later. Although liver function tests normalized and the CMV viral load significantly decreased after 6 weeks of anti-viral treatment, the infant was treated with ganciclovir or valganciclovir for a total of 6 months.

## DISCUSSION

This case describes an extremely premature infant who developed necrotizing enterocolitis and liver disease secondary to CMV and IFALD. The patient also developed a severe EFAD after lipids were held because of renal failure and HTG. This case highlights common dilemmas in neonatal lipid management. When should ILE be withheld? What is the optimal ILE and dose? How should the risks for HTG and EFAD be balanced?

HTG occurs frequently in premature infants receiving ILE because of decreased triglyceride clearance. The degree of HTG depends on lipoprotein lipase activity and correlates with low birth weight, young gestational age, sepsis, stress, and ILE dose. Although a maximum triglyceride threshold is not established in premature infants, a triglyceride level <250 mg/dL is generally well-tolerated, and HTG is usually transient. Side effects, such as pneumonitis, pancreatitis, infection, neurologic changes, and fat overload syndrome, are rare with concentrations <500 mg/dL (2). Lipid minimization may help avoid these rare complications. However, EFAD can quickly develop in premature infants because of limited fat stores and increased demands. The consequences of EFAD are serious, including growth failure and neurodevelopmental impairment. Ironically, EFAD can also exacerbate HTG because of de novo lipogenesis and fat mobilization from adipose stores. EFAD is diagnosed when the T:T (Mead acid:arachidonic acid) exceeds 0.05 in infants (1). When the supply of the ω-6 essential fatty acid, linoleic acid (LA), is insufficient; the production of arachidonic acid (ARA), a tetraene, decreases. As a result, the enzymes that metabolize LA to ARA convert the ω-9 fatty acid, oleic acid, to Mead acid, a triene.

Several ILE formulations exist and vary by oil source and phytosterol and vitamin E content (3) (See Supplemental Digital Content Table, http://links.lww.com/PG9/A25). One hundred percent soybean ILE contains a high concentration of LA and phytosterols but a low concentration of γ-tocopherol. Although this ILE provides the essential fatty acids, LA is proinflammatory and contributes to hepatic injury (4). In contrast, 100% fish oil ILE contains ω-3 fatty acids, docosahexaenoic acid (DHA) and eicosapentaenoic acid (EPA), a negligible amount of phytosterols, and α-tocopherol. DHA and EPA decrease inflammation, enhance triglyceride clearance, and help treat IFALD (4–6). Moreover, α-tocopherol and the lack of phytosterols protect against and treat IFALD. One hundred percent fish oil is the only ILE approved by the Food and Drug Administration for the management of IFALD (6). Although very small amounts of LA and ALA are present in fish oil ILE, the downstream fatty acid metabolites are sufficient to prevent EFAD (5). Finally, mixed oil ILE, a combination of soybean, olive, and fish oils along with medium-chain triglycerides, contains a more balanced ω-6:ω-3 ratio and is used off-label in infants. The minimum ILE for most premature infants to prevent an EFAD is approximately 0.5–1 and 1 g/kg/d for soybean and 100% fish oil ILE, respectively (7). For mixed oil ILE with fish oil, higher doses are required. To prevent an EFAD with mixed oil ILE, approximately 2.2–3 g/kg/d are required, and the dose depends on the infant’s gestational age and milk intake. Further research is needed to determine the optimal dose of ILE to promote adequate growth, organogenesis, and neurodevelopment. In this case, a severe EFAD was caused by withholding ILEs for over a week and suboptimal dosing of a mixed oil ILE with fish oil.

A 100% fish oil ILE successfully treated this infant’s HTG, EFAD, and IFALD. Although the typical dose of 100% fish oil ILE is 1 g/kg/d, the ILE was prescribed at 1.5 g/kg/d because of growth failure, lack of enteral nutrition, and a severe EFAD (6, 8). While ALA, DHA, and EPA concentrations increased over time, LA and ARA concentrations remained low. The implications of this fatty acid profile are unclear. Deficiencies in LA and ARA have been linked to poor growth and neurodevelopmental impairment (9). In a small randomized controlled trial, neonates receiving 100% fish oil ILE had similar neurodevelopmental outcomes when compared with those who received 100% soybean oil ILE. However, this study was terminated early and underpowered for this outcome (10). Elevated EPA levels have been reported to be associated with bleeding, although this remains controversial (5). More research is needed to elucidate how these fatty acid alterations, particularly with a normal T:T, affect clinical outcomes.

Although our infant’s growth stabilized, growth failure and microcephaly persisted. This was most likely secondary to extreme prematurity, protein-energy malnutrition, intestinal failure, EFAD, CMV, and lung disease. The infant’s slow response to 100% fish oil ILE was attributed to prematurity, CMV, a conjugated bilirubin >5 mg/dL at the initiation of 100% fish oil ILE, and a prolonged fasting state. CMV played an important role in this infant’s cholestasis, HTG, poor growth, and thrombocytopenia. Of note, infants with cholestasis secondary to viral infections are generally excluded from receiving 100% fish oil ILE (11).

In conclusion, this case highlights that the practice of ILE withholding and ILE dose restriction is not appropriate for premature infants. ILEs should be given consistently at appropriate doses to prevent an EFAD. The optimal type and dose of ILE depends on the patient’s age, prematurity and liver status, and nutritional goals. In this case, a 100% fish oil ILE dosed at 1.5 g/kg/d safely and effectively treated the infant’s HTG, EFAD, and IFALD.

## Supplementary Material


