# Supplementary material for: Essential Fatty Acid Deficiency in an Extremely Premature Infant With Intestinal Failure
Source: JPGN Rep. 2021 Apr 12;2(2):e063. doi: 10.1097/PG9.0000000000000063 (PMC10191592; doi:10.1097/PG9.0000000000000063)
Supplement: Supplementary file 1 [file pg9-2-e063-s001.pdf]

**Supplementary Table. Composition of intravenous lipid emulsions.[3]**

| Component | Soy ILE | Mixed ILE | Fish ILE |
|---|---|---|---|
| Oil Source (%) | | | |
| Soybean oil | 100 | 30 | 0 |
| Coconut oil | 0 | 30 | 0 |
| Olive oil | 0 | 25 | 0 |
| Fish oil | 0 | 15 | 100 |
| Fatty acid (% by weight) | | | |
| Linoleic acid | 54.7 | 17.5 | 1.5 |
| α-Linolenic acid, | 6.7 | 2.3 | 1.1 |
| Arachidonic acid | 0 | 0.5 | 0.2-2.0 |
| Docosahexaenoic acid | 0 | 1.0-3.5 | 14.0-27.1 |
| Eicosapentaenoic acid | 0 | 1.0-3.5 | 13.0-26.0 |
| ω-6:ω-3 ratio | 7:1 | 2.5:1 | 1:8 |
| Phytosterol (mg/L) | 423.1 | 170.6 | 2.4 |
| Vitamin E (mg/L) | 0 | 164.5 | 230.0 |
